# Mandibular Canal Morphometry and Anatomical Variations Using Cone-Beam Computed Tomography in a North Indian Population: A Retrospective Study

**DOI:** 10.7759/cureus.117407

**Published:** 2026-10-03

**Authors:** Nisha Dua, Rajesh Kumar Gupta, Swarnika Jain, Mamta Singh, Nisha Singh, Nishtha Bhutani

**Affiliations:** 1 Department of Oral Medicine and Radiology, Swami Devi Dyal Hospital and Dental College, Panchkula, IND; 2 Department of Oral Medicine and Radiology, ITS Dental College, Greater Noida, IND

**Keywords:** accessory mental foramen, anatomic variation, cone-beam computed tomography, mandible, mandibular canal

## Abstract

Introduction

The present study aimed to evaluate the morphology, dimensions, anatomical variations, and spatial characteristics of the mandibular canal (MC) in a North Indian population using cone-beam computed tomography (CBCT). The associations between MC characteristics and sex, age, and laterality were also assessed to identify clinically relevant patterns of anatomical variation.

Materials and methods

This retrospective database observational study was conducted using 400 CBCT scans of adults aged ≥18 years between June 2024 and May 2025. The scans were evaluated using multiplanar and curved planar reconstructions. MC course, length, vertical and horizontal diameters, buccolingual position, anterior loop, bifid and trifid canals, and accessory mental foramina were assessed. Two calibrated observers independently evaluated the scans, with repeated assessments performed to establish measurement reliability. Differences according to sex and side, as well as associations with age, were analyzed using generalized estimating equations (GEE) to account for bilateral observations. Multinomial logistic GEE were used for categorical canal patterns. Statistical significance was set at p < 0.05.

Results

Among the 400 participants, 220 were males and 180 were females, with a mean age of 42.6 ± 12.1 years. Males demonstrated significantly greater canal length, vertical diameter, horizontal diameter, and anterior loop length than females. Bifid canals were significantly more common in males than in females. The ascending canal morphology was the most common pattern, followed by catenary and straight configurations, with no significant sex differences. No significant right-left differences were observed in canal dimensions or anatomical variants. The canal was predominantly centrally positioned buccolingually across all tooth regions, ranging from 67.5% in the second premolar region to 76.0% in the third molar region. Increasing age was significantly associated with reduced canal length, whereas other measured characteristics showed no significant age-related associations.

Conclusion

MC anatomy demonstrates substantial morphological variability, with significant differences related to sex and age but limited bilateral asymmetry. CBCT assessment provides a detailed characterization of canal dimensions, course, and anatomical variants. Recognition of these variations is important for individualized preoperative evaluation and for minimizing neurovascular complications during mandibular surgical procedures.

## Introduction

The mandibular canal (MC) houses the inferior alveolar neurovascular bundle and represents one of the most clinically significant anatomical structures encountered during mandibular surgical procedures, including third molar extraction, implant placement, orthognathic surgery, and regional anesthesia [1]. Iatrogenic injury to the contents of this canal can result in permanent paresthesia, dysesthesia, or hemorrhagic complications, making precise preoperative localization of the canal and its surrounding osseous architecture indispensable for safe clinical practice [2].

Cone-beam computed tomography (CBCT) has emerged as the imaging modality of choice for evaluating the MC, offering high-resolution, three-dimensional, multiplanar visualization that overcomes the superimposition and magnification errors inherent in conventional panoramic radiography [1]. A recent CBCT-based investigation in a Chilean subpopulation demonstrated that a detailed morphometric assessment of canal length, its proximity to root apices, and surrounding cortical bone thickness can help establish standardized reference values applicable to surgical planning, given that the inferior alveolar nerve contained within the MC is prone to damage during clinical and surgical procedures in the mandible [3]. Similarly, morphometric evaluation of associated structures such as the mandibular incisive canal and mandibular lingual canal has been shown to be essential, since this anterior extension of the inferior alveolar canal contains neurovascular bundles and carries clinical significance. Variations in the location, number, and length of the mandibular lingual canal make its detection difficult with conventional radiographic evaluation, underscoring the need for three-dimensional imaging such as CBCT [4,5].

The MC also displays considerable anatomical variability, most notably in the form of bifid or accessory canals. A systematic review and meta-analysis pooling data from in situ, panoramic, and CBCT assessments reported that the frequency of MC variations differed markedly by imaging modality, with prevalence rates of 6.46% for in situ assessment, 4.20% for panoramic radiography, and 16.25% for CBCT, reinforcing the superior diagnostic sensitivity of CBCT [6]. In a European adult cohort, bifid canal variants were identified in 28.8% of the sides and 50.3% of the patients, with bilateral involvement noted in 7.3% of the patients [7]. Similarly, a CBCT-based cross-sectional study reported a bifid MC prevalence of approximately 10%, with a marked male predominance [8]. Alveolar bone remodeling further complicates canal morphology, as CBCT comparisons of dentulous and edentulous mandibular sides have shown measurable differences in canal position and surrounding bone dimensions following tooth loss, particularly when edentulous posterior sides are compared with dentate contralateral sides in patients who have lost all molar teeth [9].

Population-specific data remain particularly important, as the anatomical parameters of the MC show substantial ethnic and geographic variability. In India, a large orthopantomogram-based study across four geographic zones found bifid MCs in only 135 of 5800 digital orthopantomograms, corresponding to a prevalence of 2.3%, which is considerably lower than CBCT-based estimates, highlighting the diagnostic limitations of 2D imaging [10]. Likewise, a regional assessment of the mandibular incisive canal in an Indian cohort found the structure in 71.66% of CBCT scans, with 48.33% showing bilateral canals [11]. Although CBCT studies have reported MC variations in India, existing North Indian data are limited by small samples or incomplete variation classification. Given substantial ethnic and geographic variability, population-specific morphometric and variation data remain essential.

Despite this growing body of evidence, morphometric data specific to the North Indian population remain limited, and existing Indian studies have relied predominantly on panoramic radiography rather than CBCT. Notably, prior North Indian CBCT investigations have addressed lingual foramina and anterior mandibular neurovascular anatomy, leaving the diameter, position, and branching patterns of the posterior MC inadequately documented. This gap is clinically consequential, as MC morphology exhibits substantial ethnic and geographic variability, and population-specific data are essential for safe implant placement, third molar surgery, and anesthetic planning. This retrospective study aimed to comprehensively evaluate the morphometric dimensions and anatomical variations of the MC using CBCT in a North Indian population, thereby generating population-specific reference data to enhance surgical safety and treatment planning.

## Materials and methods

Study design and setting

This retrospective observational study was conducted in the Department of Oral Medicine and Radiology, Swami Devi Dyal Hospital and Dental College, Panchkula, over a period of one year (June 2024-May 2025). This study utilized pre-existing CBCT scans retrieved from the departmental digital imaging archive, which were originally acquired for diagnostic and treatment-planning purposes unrelated to the present research question (e.g., implant planning, impacted third molar assessment, endodontic evaluation, or orthodontic work-up). The scans were consecutively included if they met the eligibility criteria (Figure 1).

**Figure 1:**
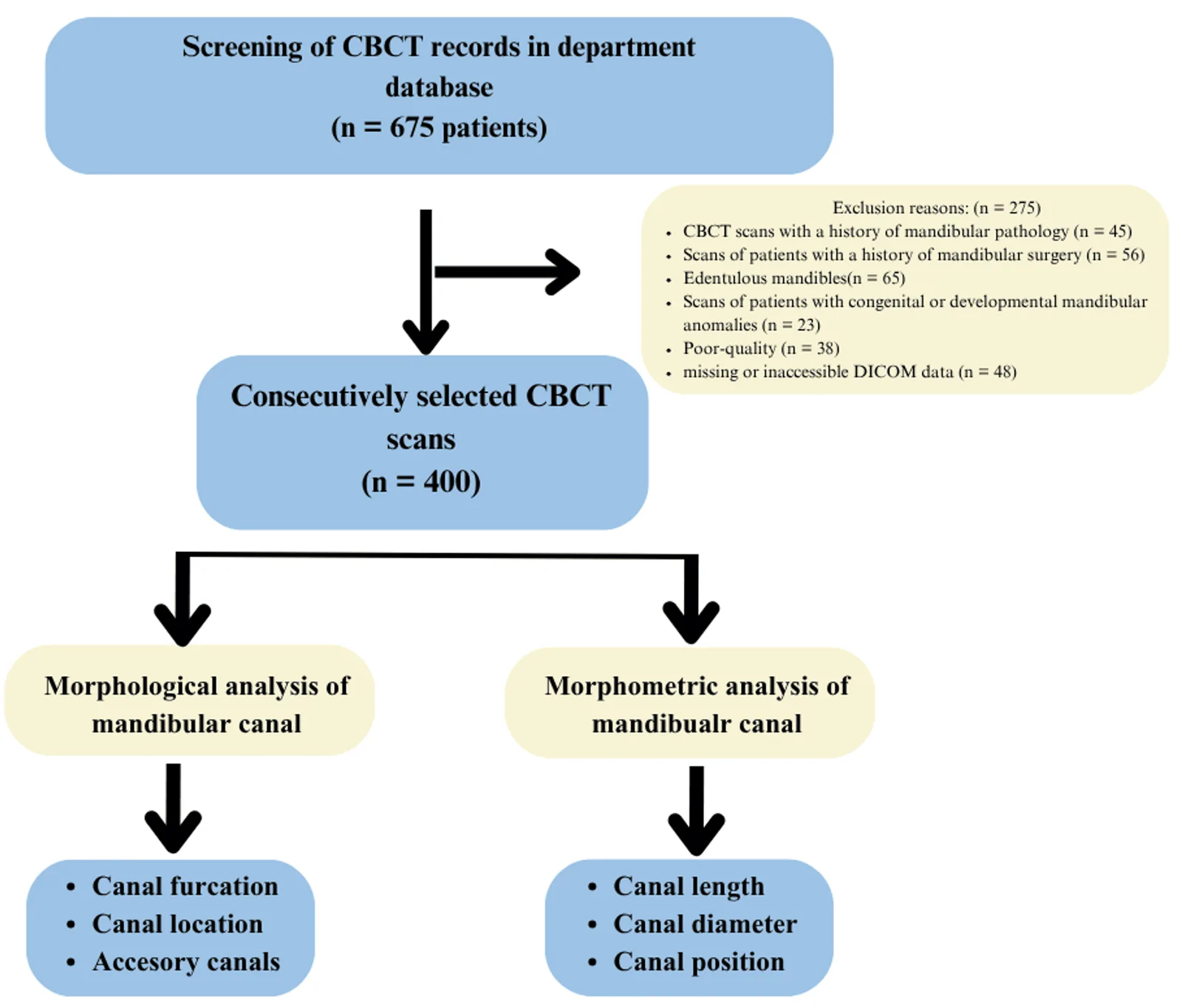
Patient selection flowchart. Image created using Canva (Canva Pty Ltd, Australia). CBCT: Cone-Beam Computed Tomography; DICOM: Digital Imaging and Communications in Medicine.

Ethical considerations

Institutional ethics committee approval (Ref. no.: SDDHDC/IEC/May2024/014, dated 13-05-2024) was obtained prior to the commencement of the study. As the study involved a retrospective analysis of pre-existing, anonymized radiographic records, a waiver of individual informed consent was granted by the committee in accordance with institutional policy for retrospective imaging-based research. The study was conducted in strict adherence to the ethical principles of the Declaration of Helsinki (as revised) for research involving human data. Patient confidentiality was maintained by assigning each CBCT record a unique numerical code at the time of retrieval; no personally identifiable information (name, contact details, or treating clinician) was accessed or recorded during data extraction or analysis.

Sample size estimation

Based on the cumulative prevalence of 62% variations of the MC documented in a prior study [12], the sample size was calculated. To estimate the proportion of a single population, the standard formula was used as follows:

$n = \frac{Z^2 \times p \times (1-p)}{d^2}$

where n is the required sample size, Z is the standard normal deviation corresponding to a 95% confidence level (1.96), p is the expected prevalence of bifid canals obtained from the reference study, and d is the allowable margin of error (5%). Using these parameters, the minimum calculated sample size was 363 CBCT scans. To account for approximately 10% potential data errors, incomplete records, or exclusions, the sample size was increased to 400 CBCT scans. Therefore, 400 CBCT images were included in this retrospective study.

Inclusion and exclusion criteria

The inclusion and exclusion criteria for record selection are summarized in Table 1.

**Table 1:** Inclusion and exclusion criteria. CBCT: Cone-Beam Computed Tomography; DICOM: Digital Imaging and Communications in Medicine.

Inclusion criteria	Exclusion criteria
CBCT scans of patients aged 18 years or older at the time of imaging.	CBCT scans of patients with a history of mandibular pathology (cysts, tumors, osteomyelitis, or fibrous dysplasia) or previous trauma or fracture involving the body or ramus of the mandible.
Scans belonging to patients native to and permanently residing in the North Indian region, based on self-reported or documented regional origin in patient records.	Scans of patients with a history of mandibular surgery, orthognathic surgery, or bone grafting on the side being evaluated.
Scans with an adequate field of view encompassing the entire mandibular body bilaterally, from the mandibular symphysis to the ramus, with a clearly traceable mandibular canal.	Edentulous mandibles or mandibles with extensive alveolar bone resorption that precludes identification of standard anatomical landmarks.
Scans of diagnostic quality, free from significant motion artifacts, metal or beam-hardening artifacts obscuring the mandibular canal, or truncation of the region of interest.	Scans of patients with congenital or developmental mandibular anomalies, such as cleft-related deformities or hemifacial microsomia.
Scans of dentate or partially dentate mandibles in the posterior region, with at least one intact molar or premolar segment, allowing standardized anatomical reference points to be established.	Scans with incomplete demographic details or missing or inaccessible DICOM data.

CBCT acquisition protocol

All CBCT scans included in the study were acquired using a designated CBCT unit, Vatech PaX-i3D Smart (Vatech India Pvt. Ltd., New Delhi, India), installed in the Department of Oral Medicine and Radiology. The scans were obtained according to the manufacturer’s standardized exposure protocol using a tube voltage of 90 kVp and a tube current of 8 mA, adjusted according to the patient’s build. A field of view (FOV) of 12 × 9 cm was used for the standard mandibular protocol, and scans with a smaller FOV that did not encompass the complete course of the MC were excluded. The isotropic voxel size ranged from 0.20 to 0.40 mm, while the scan/exposure time varied from approximately 18 to 20 s depending on the selected resolution mode.

The patients were positioned in a seated or standing position with the Frankfort horizontal plane parallel to the floor and stabilized using head-positioning guides and a bite block, in accordance with the standard departmental protocol. Image reconstruction was performed using the manufacturer’s proprietary Ez3D-i Imaging Software (Vatech, Hwaseong-si, Gyeonggi-do, Republic of Korea), following which the raw Digital Imaging and Communications in Medicine (DICOM) datasets were exported for independent analysis. All measurements in the present study were carried out on exported DICOM files using image analysis software (Ez3D-i Imaging Software), which permitted multiplanar reconstruction (axial, coronal, and sagittal) and cross-sectional/panoramic curved reformatting along the dental arch.

Image analysis and calibration

Two independent, calibrated observers (a postgraduate trainee and a senior faculty member with over five years of CBCT interpretation experience from the Department of Oral Medicine and Radiology) evaluated all scans in a blinded, randomized sequence, unaware of patient identity and each other's measurements. Prior to formal data collection, a calibration exercise was conducted on 20 randomly selected scans (not included in the final dataset) to standardize measurement techniques and landmark identification. Inter-observer and intra-observer reliabilities were assessed using the intraclass correlation coefficient (ICC), with an ICC value ≥0.80 considered indicative of acceptable reliability. A second reading was performed by each observer on the same 20 scans after a two-week interval to compute intra-observer agreement. In cases of measurement discrepancy exceeding a predefined threshold (>0.5 mm for linear measurements or disagreement in categorical classification), a consensus reading was obtained through joint re-evaluation.

Parameters assessed and measurement protocol

The MC course/pattern was traced from the mandibular foramen to the mental foramen on sagittal and panoramic curved reconstructions and classified according to its vertical course as ascending, catenary (curved), or straight (linear), based on established radiographic classification criteria [13].

Canal length was measured as the linear distance (mm) along the curved trajectory of the canal from the mandibular foramen (entry point on the medial ramal surface) to the mental foramen (exit point), traced using the curved multiplanar reconstruction tool, and recorded separately for the right and left sides (Figure 2).

**Figure 2:**
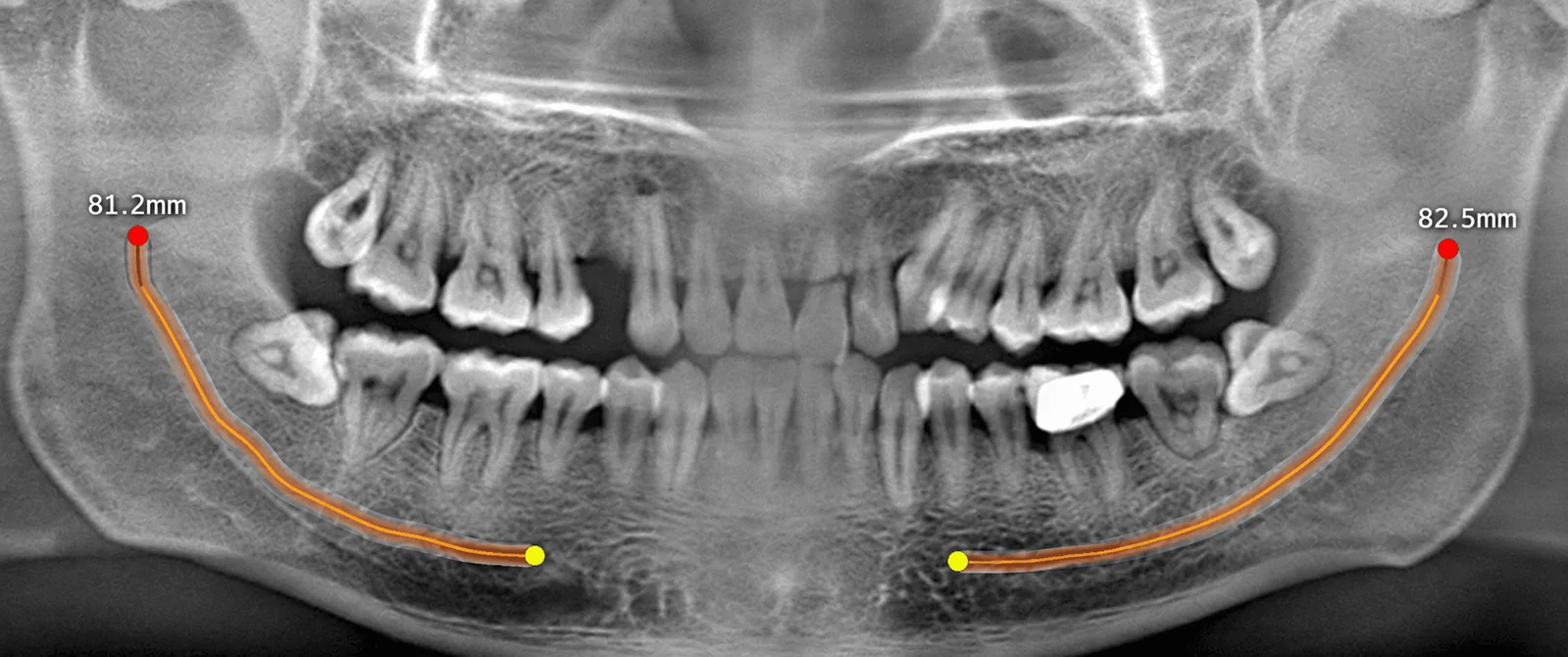
CBCT panoramic reconstruction demonstrating bilateral measurement of the mandibular canal length, measuring 81.2 mm on the right side and 82.5 mm on the left side. Mandibular foramen (red dot); mental foramen (yellow dot). CBCT: Cone-beam computed tomography.

Canal diameter (horizontal and vertical) was measured on cross-sectional images at three standardized reference points along the canal course (corresponding to the regions of the mandibular first molar, second molar, and mental foramen), using the horizontal and vertical internal diameters (mm) of the radiolucent canal outline, and mean values were calculated for each side.

Canal position (buccal, lingual, or central) was assessed on cross-sectional CBCT images at the second premolar, first molar, second molar, and third molar regions. At each site, the position of the MC was determined relative to the buccolingual midpoint of the mandibular body and categorized as buccal, lingual, or central based on which cortical plate the canal lay closer to.

Bifid/trifid canals on sequential axial, coronal, and cross-sectional images were identified as the presence of two (bifid) or three (trifid) distinct radiolucent canal pathways within the mandibular body, with the location (ramus, body, or both) documented (Figure 3).

**Figure 3:**
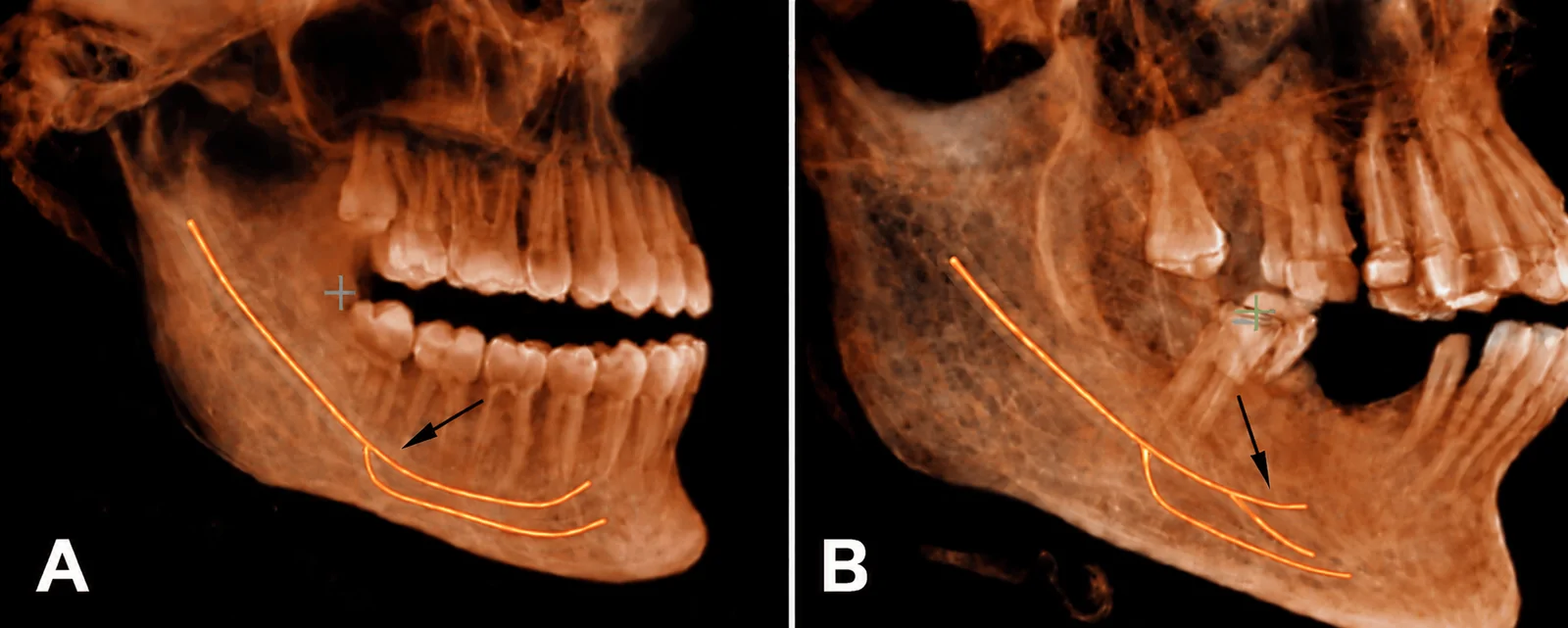
CBCT images demonstrating variations of the mandibular canal. (A) Bifid mandibular canal and (B) Trifid mandibular canal, indicated by black arrows. CBCT: Cone-beam computed tomography.

Accessory foramen was recorded as the presence of additional radiolucent foraminal openings distinct from the primary mental foramen or mandibular foramen, identified on 3D volume-rendered and cross-sectional images, with the number and approximate location (relative to the adjacent tooth or root) documented (Figure 4).

**Figure 4:**
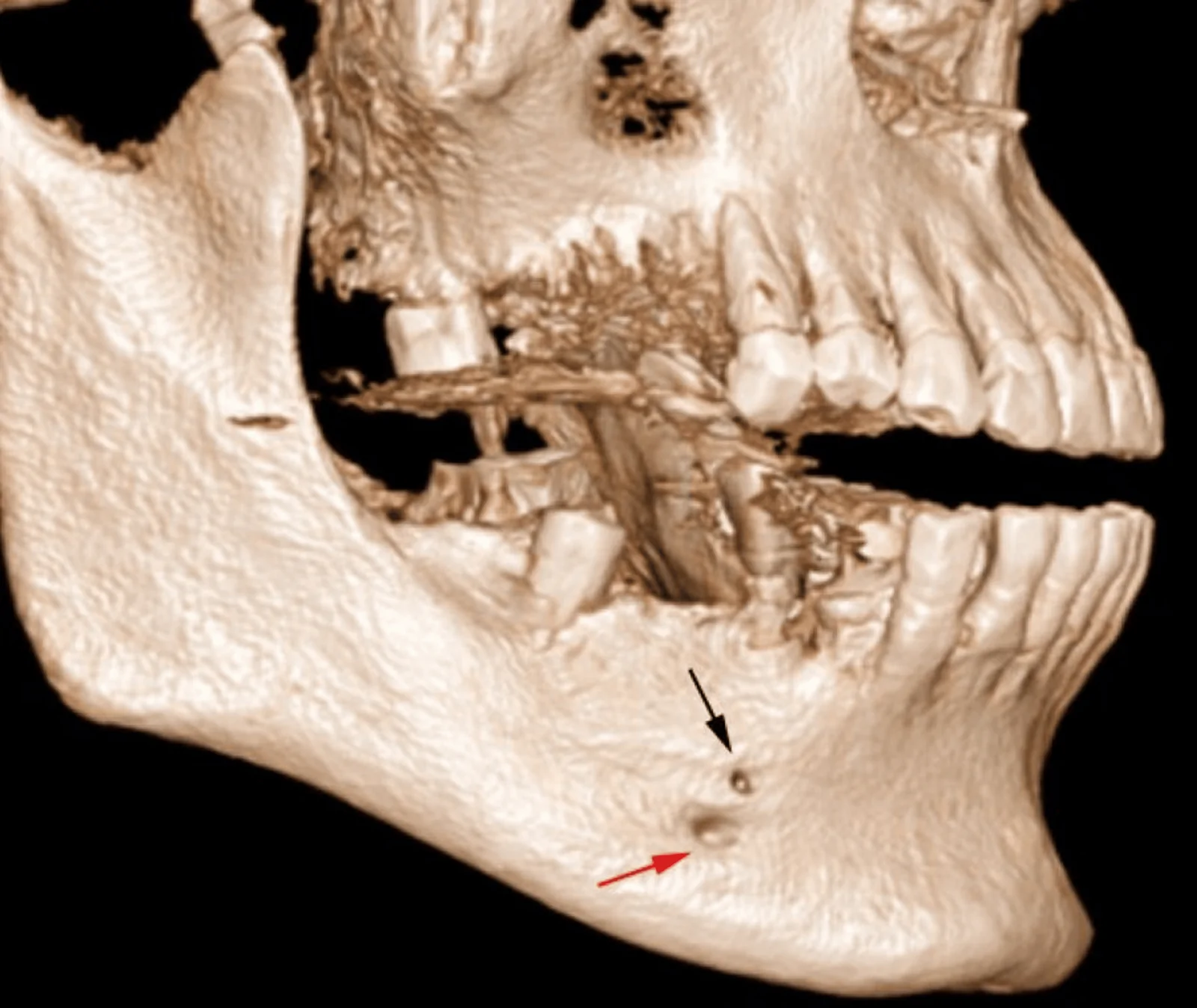
CBCT 3D reconstruction demonstrating the mental foramen (red arrow) and accessory mental foramen (black arrow) on the lateral aspect of the mandible. CBCT: Cone-beam computed tomography.

The presence of an anterior loop of the mental nerve was assessed on sagittal cross-sectional images anterior to the mental foramen, and when present, its length was measured (mm) as the linear distance from the anterior-most extent of the loop to the posterior margin of the mental foramen.

All linear measurements were recorded to the nearest 0.01 mm using the built-in calibrated measurement tool of the viewing software and cross-verified against the known voxel dimensions of each scan.

Data recording and management

Demographic information (age and sex) was obtained from the electronic dental records accompanying each CBCT scan. Dental status (dentulous mandibular jaw) was also recorded from the dental records and confirmed on the CBCT images. Data for each scan were entered into a pre-structured, coded Microsoft Excel worksheet, including demographic variables (age and sex) and all radiographic parameters listed above, which were recorded separately for the right and left sides. Data were periodically cross-checked for entry errors before statistical analysis.

Statistical analysis

Statistical analysis was performed using SPSS software version 25 (IBM Corp., Armonk, NY, USA). Descriptive statistics were expressed as mean ± standard deviation for continuous variables and as frequencies with percentages for categorical variables. An independent-samples t-test was used to compare age between sexes, whereas the chi-square test was used to assess differences in age-group distribution. Generalized estimating equations (GEE) were employed to evaluate sex-related and side-related differences in MC parameters, accounting for within-subject correlation from bilateral measurements. GEE with multinomial logistic regression was used to analyze canal patterns and buccolingual orientation. Age associations were examined using GEE with continuous age as a predictor. Statistical significance was set at p < 0.05, and effect sizes (Cohen's d, Cramer's V, and odds ratios) were calculated, where applicable.

## Results

The study population comprised 400 participants (220 males (55%) and 180 females (45%)) with a mean age of 42.6 ± 12.1 years. Males were significantly older than females (43.8 ± 12.3 vs. 41.1 ± 11.7 years; t = 2.24, p = 0.026), with a small effect size (Cohen's d = 0.23). The age group distribution showed that the 31-40 years age group was the most represented (26.5%), followed by the 41-50 years age group (25.8%). No significant difference was observed in the age group distribution between sexes (χ² = 2.31, p = 0.511, Cramer's V = 0.076), indicating comparable age group proportions across males and females despite the small but significant difference in mean age. A total of 800 MCs were observed, with 440 (55.0%) in males and 360 (45.0%) in females. The sex distribution of MCs differed significantly (p = 0.047), with a small effect (Cohen’s w = 0.10) (Table 2).

**Table 2:** Demographic characteristics of the study population. Data are presented as mean ± SD for continuous variables and as frequency (n) and percentage (%) for categorical variables. An independent-samples t-test was used to compare mean age between males and females, and the chi-square test was used to compare age-group distribution between sexes. A chi-square goodness-of-fit test was used to determine whether the sex distribution differed from the expected 50:50 distribution. Effect sizes were expressed as Cohen’s d for continuous variables, Cramer's V for age-group distribution, and Cohen's w for the goodness-of-fit test. *Statistical significance was set at p < 0.05. MC: Mandibular canal.

Demographic variable	Category	Overall (N = 400)	Male (n = 220)	Female (n = 180)	Test value	p-value	Effect size
Age, mean ± SD	Years	42.6 ± 12.1	43.8 ± 12.3	41.1 ± 11.7	t = 2.24	0.026*	Cohen's d = 0.23
Age group, n (%)	18-30 years	78 (19.5)	39 (17.7)	39 (21.7)	χ² = 2.31	0.511	Cramer's V = 0.076
	31-40 years	106 (26.5)	55 (25.0)	51 (28.3)			
	41-50 years	103 (25.8)	61 (27.7)	42 (23.3)			
	51-60 years	72 (18.0)	43 (19.5)	29 (16.1)			
	>60 years	41 (10.3)	22 (10.0)	19 (10.6)			
Number of MCs observed, n (%)	Units	800 (100)	440 (55)	360 (45)	χ² = 8.00	0.047*	Cohen's w = 0.10

Males demonstrated significantly larger MC dimensions than females across all measured parameters. Canal length was significantly greater in males (82.4 ± 9.8 mm vs. 79.8 ± 9.5 mm; β = 2.60, p = 0.007, Cohen's d = 0.27), as were vertical diameter (2.31 vs. 2.22 mm; β = 0.09, p = 0.006, d = 0.28) and horizontal diameter (2.54 vs. 2.45 mm; β = 0.09, p = 0.019, d = 0.24). The anterior loop length was also notably longer in males (2.91 vs. 2.74 mm; β = 0.17, p = 0.043, d = 0.20). Regarding anatomical variations, bifid MCs were significantly more frequently found in males (OR = 1.51, p = 0.040), while trifid MCs (OR = 1.69, p = 0.233), accessory mental foramina (OR = 1.37, p = 0.187), and anterior loops (OR = 1.44, p = 0.075) showed a higher but nonsignificant prevalence in males (Table 3).

**Table 3:** Sex-related differences in mandibular canal morphology and morphometric parameters using GEE. Data are presented as mean ± SD for continuous variables and as frequency (n) and percentage (%) for categorical variables. GEE were used to account for the within-subject correlation of bilateral mandibular observations. Regression coefficients (β) with 95% CI and Wald χ² statistics were reported for continuous outcomes, whereas OR with 95% CI and Wald χ² statistics were reported for binary outcomes. Cohen’s d was used as the effect size for continuous variables. Statistical significance was set at p < 0.05. GEE: Generalized estimating equations; β: Regression coefficient; χ²: Chi-square; d: Cohen’s d. Bilateral mandibular canal characteristics are based on 800 canal observations from 400 participants (two observations per participant).

Outcome	Male (n = 440 canals)	Female (n = 360 canals)	GEE estimate (β/OR)	95% CI	Wald χ²	p-value	Effect size
Canal length (mm)	82.4 ± 9.8	79.8 ± 9.5	β = 2.60	0.70 to 4.50	7.16	0.007*	Cohen's d = 0.27
Vertical canal diameter (mm)	2.31 ± 0.34	2.22 ± 0.31	β = 0.09	0.03 to 0.15	7.48	0.006*	Cohen's d = 0.28
Horizontal canal diameter (mm)	2.54 ± 0.39	2.45 ± 0.37	β = 0.09	0.01 to 0.17	5.52	0.019*	Cohen's d = 0.24
Anterior loop length (mm)	2.91 ± 0.86	2.74 ± 0.81	β = 0.17	0.01 to 0.33	4.08	0.043*	Cohen's d = 0.20
Bifid canal, n (%)	78 (17.7)	48 (13.3)	OR = 1.51	1.01 to 2.26	4.2	0.040*	OR = 1.51
Trifid canal, n (%)	16 (3.6)	8 (2.2)	OR = 1.69	0.70 to 4.08	1.42	0.233	OR = 1.69
Accessory mental foramen, n (%)	56 (12.7)	36 (10.0)	OR = 1.37	0.85 to 2.19	1.74	0.187	OR = 1.37
Anterior loop, n (%)	146 (33.2)	104 (28.9)	OR = 1.44	0.97 to 2.14	3.18	0.075	OR = 1.44

The ascending pattern was the most frequently observed canal configuration (41.3%), followed by the catenary (33.3%) and straight (25.5%) patterns. In males, an ascending pattern was observed in 43.2% of the MCs, compared with 38.9% in females. The catenary pattern was slightly more common in females (33.9%) than in males (32.7%), whereas the straight pattern was more common in females (27.2%) than in males (24.1%). However, statistical analysis using GEE revealed no significant sex differences in canal pattern distribution. Compared with the ascending pattern (reference), the odds of the catenary pattern (OR = 0.95, p = 0.733) and straight pattern (OR = 0.85, p = 0.306) did not differ significantly between males and females (Table 4).

**Table 4:** Mandibular canal pattern according to sex using GEE. Data are presented as frequencies (n) and percentages (%). Multinomial logistic GEE were used to assess the association between sex and mandibular canal pattern while accounting for within-subject bilateral correlation. OR with 95% CI and Wald χ² statistics were reported. A p-value < 0.05 was considered statistically significant. Percentages for bilateral mandibular canal characteristics are based on 800 canal observations from 400 participants (two observations per participant). GEE: Generalized estimating equations; χ²: Chi-square.

Canal pattern	Overall (n = 800 canals), n (%)	Male (n = 440 canals), n (%)	Female (n = 360 canals), n (%)	GEE-estimated OR	95% CI	Wald χ²	p-value
Ascending	330 (41.3)	190 (43.2)	140 (38.9)	Reference	-	-	-
Catenary	266 (33.3)	144 (32.7)	122 (33.9)	OR = 0.95	0.70-1.29	0.12	0.733
Straight	204 (25.5)	106 (24.1)	98 (27.2)	OR = 0.85	0.61 to 1.18	1.05	0.306

A comparative analysis of the right and left MCs showed no significant bilateral differences in any of the measured parameters. Canal length was comparable between the right (81.1 ± 9.7 mm) and left (80.7 ± 9.6 mm) sides (β = 0.40, p = 0.110, Cohen's d = 0.04). Similarly, vertical diameter (2.28 vs. 2.26 mm; β = 0.02, p = 0.182, d = 0.06), horizontal diameter (2.51 vs. 2.49 mm; β = 0.02, p = 0.267, d = 0.05), and anterior loop length (2.86 vs. 2.80 mm; β = 0.06, p = 0.153, d = 0.07) showed no significant asymmetry. Anatomical variations, including bifid MCs (OR = 1.04, p = 0.842), trifid MCs (OR = 1.19, p = 0.680), accessory mental foramina (OR = 1.10, p = 0.663), and anterior loops (OR = 1.05, p = 0.764), also demonstrated no significant side predilection, confirming the bilateral symmetry of MC morphology (Table 5).

**Table 5:** Right- versus left-sided mandibular canal characteristics. Data are presented as mean ± SD for continuous variables and as frequency (n) and percentage (%) for categorical variables. GEE were used to assess right-left differences while accounting for within-subject correlation. Regression coefficients (β) with 95% CI and Wald χ² statistics were reported for continuous variables, whereas OR with 95% CI and Wald χ² statistics were reported for categorical variables. Cohen’s d was used as the effect size for continuous variables. A p-value < 0.05 was considered statistically significant. GEE: Generalized estimating equations; β: Regression coefficient; χ²: Chi-square; d: Cohen’s d. Percentages for bilateral mandibular canal characteristics are based on 800 canal observations from 400 participants (two observations per participant).

Outcome	Right side (n = 400 canals)	Left side (n = 400 canals)	GEE estimate (β/OR)	95% CI	Wald χ²	p-value	Effect size
Canal length (mm)	81.1 ± 9.7	80.7 ± 9.6	β = 0.40	-0.09 to 0.89	2.56	0.11	Cohen's d = 0.04
Vertical canal diameter (mm)	2.28 ± 0.33	2.26 ± 0.32	β = 0.02	-0.01 to 0.05	1.78	0.182	Cohen's d = 0.06
Horizontal canal diameter (mm)	2.51 ± 0.38	2.49 ± 0.37	β = 0.02	-0.02 to 0.06	1.23	0.267	Cohen's d = 0.05
Anterior loop length (mm)	2.86 ± 0.85	2.80 ± 0.83	β = 0.06	-0.02 to 0.14	2.04	0.153	Cohen's d = 0.07
Bifid canal, n (%)	64 (16.0)	62 (15.5)	OR = 1.04	0.71 to 1.52	0.04	0.842	OR = 1.04
Trifid canal, n (%)	13 (3.3)	11 (2.8)	OR = 1.19	0.53 to 2.67	0.17	0.68	OR = 1.19
Accessory mental foramen, n (%)	48 (12.0)	44 (11.0)	OR = 1.10	0.71 to 1.72	0.19	0.663	OR = 1.10
Anterior loop, n (%)	127 (31.8)	123 (30.8)	OR = 1.05	0.78 to 1.41	0.09	0.764	OR = 1.05

Central orientation was the most predominant buccolingual position across all tooth regions, ranging from 67.5% in the second premolar to 76.0% in the third molar. No significant sex differences were observed in orientation patterns at any tooth level. At the second premolar, buccal orientation showed a comparable prevalence between males (22.5%) and females (20.8%; OR = 1.10, p = 0.590), whereas lingual orientation showed no significant difference (OR = 0.84, p = 0.462). Similarly, in the first, second, and third molars, neither buccal nor lingual orientation demonstrated a significant sex predilection (all p > 0.05). The central position consistently represented the majority orientation, indicating that the MC predominantly runs centrally through the mandible, regardless of sex or tooth region (Table 6).

**Table 6:** Buccolingual orientation of the mandibular canal according to tooth region and sex. Data are presented as frequencies (n) and percentages (%). Multinomial logistic GEE were used to compare buccolingual canal positions between sexes while accounting for the within-subject correlation of bilateral observations. OR with 95% CI and Wald χ² statistics are reported. Statistical significance was set at p < 0.05. GEE: Generalized estimating equations; χ²: Chi-square. Percentages for bilateral mandibular canal characteristics are based on 800 canal observations from 400 participants (two observations per participant).

Tooth region	Orientation	Overall (n = 800 canals), n (%)	Male (n = 440 canals), n (%)	Female (n = 360 canals), n (%)	GEE-estimated OR	95% CI	Wald χ²	p-value
Second premolar	Buccal	174 (21.8)	99 (22.5)	75 (20.8)	OR = 1.10	0.78 to 1.55	0.29	0.59
	Central	540 (67.5)	297 (67.5)	243 (67.5)	Reference	-	-	-
	Lingual	86 (10.8)	44 (10.0)	42 (11.7)	OR = 0.84	0.53 to 1.33	0.54	0.462
First molar	Buccal	146 (18.3)	79 (18.0)	67 (18.6)	OR = 0.96	0.67 to 1.37	0.06	0.807
	Central	562 (70.3)	312 (70.9)	250 (69.4)	Reference	-	-	-
	Lingual	92 (11.5)	49 (11.1)	43 (11.9)	OR = 0.92	0.59 to 1.44	0.13	0.718
Second molar	Buccal	108 (13.5)	58 (13.2)	50 (13.9)	OR = 0.94	0.62 to 1.42	0.08	0.777
	Central	592 (74.0)	328 (74.5)	264 (73.3)	Reference	-	-	-
	Lingual	100 (12.5)	54 (12.3)	46 (12.8)	OR = 0.95	0.62 to 1.46	0.05	0.823
Third molar	Buccal	76 (9.5)	40 (9.1)	36 (10.0)	OR = 0.90	0.56 to 1.45	0.19	0.663
	Central	608 (76.0)	335 (76.1)	273 (75.8)	Reference	-	-	-
	Lingual	116 (14.5)	65 (14.8)	51 (14.2)	OR = 1.05	0.70 to 1.57	0.06	0.807

Age demonstrated a significant negative association only with canal length (β = -0.08 mm per year; 95% CI: -0.14 to -0.02; p = 0.010), indicating gradual shortening of the MC with advancing age. Although there was a tendency for the vertical canal diameter to decrease with age (β = -0.002, p = 0.072), this was not statistically significant. The horizontal diameter (β = -0.001, p = 0.290) and anterior loop length (β = 0.005, p = 0.110) showed no significant age-related changes. Regarding anatomical variations, bifid MCs showed a trend toward decreased prevalence with age (OR = 0.89 per 10 years, p = 0.082), whereas trifid MCs (OR = 0.85, p = 0.140), accessory mental foramina (OR = 0.95, p = 0.498), and anterior loops (OR = 1.03, p = 0.590) demonstrated no significant age-related associations (Table 7).

**Table 7:** Association between age and mandibular canal characteristics using GEE. GEE were used to evaluate associations between age and mandibular canal characteristics while accounting for the within-subject correlation of bilateral observations. Regression coefficients (β) with 95% CI and Wald χ² statistics were reported for continuous outcomes, whereas OR with 95% CI and Wald χ² statistics were reported for categorical outcomes. For categorical outcomes, the OR represents the change associated with a 10-year increase in age. Statistical significance was set at p < 0.05. GEE: Generalized estimating equations; β: Regression coefficient; χ²: Chi-square. Bilateral mandibular canal characteristics are based on 800 canal observations from 400 participants (two observations per participant).

Outcome	Age effect (β/OR)	95% CI	Wald χ²	p-value
Canal length (mm)	β = -0.080	-0.140 to -0.020	6.72	0.010*
Vertical canal diameter (mm)	β = -0.002	-0.004 to 0.001	3.24	0.072
Horizontal canal diameter (mm)	β = -0.001	-0.003 to 0.001	1.12	0.29
Anterior loop length (mm)	β = 0.005	-0.001 to 0.011	2.56	0.11
Bifid canal	OR = 0.890	0.780 to 1.020	3.02	0.082
Trifid canal	OR = 0.850	0.680 to 1.060	2.18	0.14
Accessory mental foramen	OR = 0.950	0.820 to 1.100	0.46	0.498
Anterior loop	OR = 1.030	0.920 to 1.150	0.29	0.59

## Discussion

This retrospective CBCT study provided a comprehensive assessment of MC morphology, morphometry, and anatomical variants in 400 adults from a North Indian population.

Sex-related differences in MC dimensions

One of the most consistent findings of the present study was a greater MC length in males than in females. Mean canal length was significantly associated with sex. This observation is biologically plausible because mandibular dimensions, cortical thickness, and overall craniofacial skeletal size are generally larger in males. Importantly, this finding is consistent with recent CBCT-based evidence. A Chilean study demonstrated significantly greater MC length in men than in women, although the absolute canal lengths reported were lower than those observed in the present North Indian sample [3]. Similarly, Okiriamu A et al. reported significantly larger MC diameters in males in the second premolar, first molar, and second molar regions, supporting the present observation that the canal is morphometrically larger in males [12]. The consistency of these findings across geographically distinct populations, Chilean, Kenyan, and North Indian, suggests that sexual dimorphism in MC dimensions reflects fundamental skeletal growth patterns rather than population-specific traits. However, the absolute differences in canal length between studies may be attributable to variations in measurement protocols, including landmark selection for the mandibular foramen and mental foramen, the use of axial versus panoramic reconstruction planes, and differences in CBCT voxel size and slice thickness.

Additionally, the present study demonstrated significantly greater vertical and horizontal canal diameters in males. Recent CBCT investigations have similarly demonstrated larger MC dimensions or surrounding mandibular measurements in males [3,12]. Nevertheless, these differences should be interpreted primarily as population-level tendencies rather than as clinically reliable sex-specific reference values.

Anterior loop morphology

In the present study, anterior loops were identified in approximately one-third of the assessed hemimandibles, with a mean loop length of approximately 2.8-2.9 mm. The loop was somewhat more prevalent and longer in males, with the sex difference in loop length reaching statistical significance, whereas the difference in prevalence did not.

The prevalence observed in the present study is broadly compatible with the contemporary literature, although considerable variation between studies is expected. A systematic review and meta-analysis involving 13,743 participants and 27,075 hemimandibles reported anterior loops in 40.6% of individuals and 36.0% of hemimandibles, with an overall mean extension of approximately 2.4 mm [14]. Importantly, the meta-analysis found no significant difference in loop prevalence between males and females, although male loops were somewhat longer [14]. This agrees particularly well with the present findings: prevalence showed only a nonsignificant male predominance, whereas loop length demonstrated a significant sex-related difference.

However, other Indian studies have reported markedly different prevalence rates. A retrospective Indian CBCT study reported anterior loops in only 6.9% of hemimandibles, with loop lengths ranging from 2.4 to 6.6 mm [15]. Conversely, a South Indian comparison of panoramic radiography and CBCT reported substantially higher CBCT detection rates [16]. These discrepancies emphasize that anterior loop prevalence is strongly influenced by the operational definition used, CBCT acquisition parameters, reconstruction plane, population characteristics, and observer interpretation.

From a clinical perspective, the mean anterior loop length identified in the present investigation is particularly relevant because relatively small differences in measurement can affect implant positioning. A systematic review by Hadilou M et al. suggested considering a 2-mm safety margin around the anterior loop when planning procedures in this region [14]. Consequently, even when the anterior loop is short, it should not be disregarded during implant planning.

Bifid and trifid MCs

Bifid MCs were another important finding of this study. The present study identified bifid canals in approximately 16% of the hemimandibles, with a significant male predominance. The odds of having a bifid canal were approximately 1.5-fold higher in males. This finding is consistent with several CBCT studies demonstrating that accessory canal morphology is relatively common but highly variable among populations.

Varvara G et al. identified mandibular canal variants in 50.3% of individuals and 28.8% of hemimandibles in a 2022 European CBCT study, although their study included several types of accessory canals, rather than only bifid canals. They observed variants in 52.4% of males and 48.4% of females, indicating only a modest sex difference [7]. More recently, Menditti D et al. identified mandibular canal variations in 31% of patients and 18% of hemimandibles, again emphasizing the substantial anatomical heterogeneity [17]. A recent CBCT study also reported bifid canals as the most common variation, occurring in approximately 36.2% of patients [18]. Thus, the frequency observed in the present investigation is within the broad range reported in contemporary CBCT literature.

However, evidence regarding sex predilection remains contradictory. A CBCT study specifically examining MC bifurcation found bifurcation in 22.3% of scans but reported no significant association with sex or age; interestingly, bifurcations were numerically more common in females [19]. This finding contrasts with the significant male association observed in the present study. Such disagreements may reflect population differences, sample composition, bifurcation definitions, classification criteria, and imaging methodologies. Therefore, the present finding should be interpreted as evidence of a sex-associated tendency in this North Indian sample rather than as proof of an inherent universal male predisposition.

Trifid canals were considerably less frequent than bifid canals and showed no significant association with sex. This was expected because increasing branching complexity represents a relatively uncommon anatomical phenotype. Even an uncommon trifid configuration may contain neurovascular structures and therefore has potential implications for osteotomy, implant placement, and mandibular nerve block procedures.

MC course

The ascending canal pattern was the most common configuration, followed by the catenary and straight patterns. No significant sex-related differences were observed between groups. The predominance of an ascending/progressive course is supported by the Kenyan CBCT study by Okiriamu A et al., in which the progressive-descent type was the dominant canal configuration [12]. Although the terminology and classification systems differ between studies, both findings demonstrate that the MC frequently deviates from an idealized horizontal trajectory. The absence of sex-related differences in canal course suggests that canal configuration may be more strongly determined by individual anatomical development than by sexual dimorphism.

Buccolingual position

The MC was predominantly centrally positioned at the second premolar, first molar, second molar, and third molar levels. Nevertheless, buccal or lingual displacement has direct surgical implications in a substantial number of cases. A canal positioned closer to the buccal or lingual cortical plate may reduce the available bone corridor for implant placement and alter the risk of cortical perforation or neurovascular injury. Contemporary CBCT literature emphasizes that MC location varies among individuals and populations and should therefore be evaluated three-dimensionally rather than inferred from panoramic anatomy [3,12].

Bilateral symmetry

A notable finding was the absence of significant right-left differences in all outcome parameters. This finding supports a degree of bilateral anatomical symmetry of the MC.

This finding is concordant with the Chilean CBCT study, which found no significant difference in MC length between the right and left sides [3]. Similarly, contemporary literature on accessory canal morphology indicates that although unilateral variants may predominate, bilateral occurrence is also possible and cannot be reliably predicted from the contralateral side [7]. Therefore, the bilateral symmetry observed at the population level should not be used as justification for assuming that one side mirrors the other in an individual patient.

In the present study, the use of GEE was particularly appropriate for this outcome because bilateral observations within the same individual were correlated. By accounting for within-subject clustering, the analysis reduced the risk of falsely identifying side differences due to the inappropriate treatment of paired observations as independent.

Age-related changes

Increasing age was significantly associated with a small reduction in MC length of approximately 0.08 mm per year. However, no significant age-related association was observed for the other outcomes. Age-related shortening of the canal should be interpreted cautiously. The present study was cross-sectional; therefore, longitudinal anatomical remodeling could not be established. Age may act as a surrogate for cumulative alveolar remodeling, tooth loss, and changes in mandibular morphology rather than directly causing shortening of the canal. Previous CBCT evidence has demonstrated that tooth loss and changes in dentition can influence MC position and surrounding bone morphology [9]. More recent longitudinal CBCT studies have also demonstrated that MC dimensions and position can change during growth, particularly in younger individuals [20]. Although the latter population differed substantially from the adult cohort in the present study, this reinforces the broader concept that the MC should not be regarded as an absolutely static anatomical structure.

The absence of significant age-related associations for bifid canals, trifid canals, accessory mental foramina, and anterior loop prevalence is broadly consistent with recent evidence. The bifurcation study found no significant association between MC bifurcation and age [19]. Likewise, a recent Indian CBCT investigation reported relatively stable variation prevalence across age groups [15]. These observations suggest that most canal variants represent developmental anatomical patterns rather than changes acquired with age.

Accessory mental foramina

Accessory mental foramina were identified in approximately 12% of hemimandibles in the present study, without a significant sex or side predilection. This finding has important clinical implications because an accessory foramen may transmit additional branches of the mental nerve and vessels. The reported prevalence is somewhat higher than the pooled prevalence in a systematic review and meta-analysis of CBCT studies, which estimated accessory mental foramina in approximately 7.9% of subjects and 4.8% of hemimandibles [21]. A Saudi multicenter CBCT study reported a prevalence of approximately 10%, demonstrating that regional differences can be substantial [22]. Therefore, the higher prevalence in the present study may reflect population-specific anatomy, differences in the minimum size required to identify a foramen, and differences in CBCT resolution and interpretation.

Clinical implications

These findings have direct implications for implantology, third molar surgery, orthognathic surgery, periapical surgery, mandibular osteotomy, and regional anesthesia. The demonstrated sex-related differences in canal dimensions, relatively frequent bifid canals, anterior loops, and accessory foramina, and the presence of buccolingual positional variation collectively indicate that MC anatomy should be regarded as patient-specific. Importantly, bilateral symmetry cannot be assumed despite the absence of significant side differences at the population level. Therefore, CBCT evaluation should include systematic tracing of the canal in axial, sagittal, coronal, and curved planar reconstructions. The identification of anterior loops and accessory canals is particularly important before implant placement in the premolar/interforaminal region, whereas bifid or trifid canals should be considered during posterior mandibular surgery. These findings support individualized CBCT-based surgical planning rather than reliance on conventional two-dimensional anatomical assumptions. However, CBCT should be employed when clinically indicated rather than categorically as the imaging modality of choice for every mandibular procedure. The principle of As Low As Reasonably Achievable (ALARA) should guide decision-making, with CBCT reserved for cases in which three-dimensional information is reasonably expected to alter surgical planning or reduce the risk of neurovascular injury.

Limitations

This study has several limitations. First, its retrospective cross-sectional design prevents the determination of causal relationships, particularly regarding the observed association between increasing age and reduced canal length. Second, the sample was derived from a single-institution CBCT archive and included patients from a defined North Indian population. Consequently, these findings may not be generalizable to other ethnic or geographic populations. Third, variations in dental status, mandibular morphology, and previous tooth loss may influence canal dimensions but were not modeled as independent predictors. Finally, the study did not evaluate clinical outcomes following surgical procedures; therefore, the association between individual anatomical variants and actual neurovascular complications could not be established.

## Conclusions

This study demonstrated significant sex-related differences in MC dimensions, with males exhibiting greater canal length and diameter, while bilateral differences were not significant. The ascending canal pattern was the most common, followed by the catenary and straight patterns. Bifid canals showed a significant male predominance, whereas trifid canals, accessory mental foramina, and anterior loops were not significantly associated with sex. Canal length was significantly inversely associated with age. Overall, these findings highlight the considerable anatomical variability of the MC in a North Indian population and emphasize the importance of careful CBCT-based assessment before implant placement, mandibular surgery, and other procedures involving the inferior alveolar neurovascular bundle.
